# Comparison of deep learning with traditional models to predict preventable acute care use and spending among heart failure patients

**DOI:** 10.1038/s41598-020-80856-3

**Published:** 2021-01-13

**Authors:** Maor Lewis, Guy Elad, Moran Beladev, Gal Maor, Kira Radinsky, Dor Hermann, Yoav Litani, Tal Geller, Jesse M. Pines, Nathan l. Shapiro, Jose F. Figueroa

**Affiliations:** 1 Diagnostic Robotics Inc., Tel Aviv, Israel; 2 US Acute Care Solutions, Canton, OH USA; 3 Department of Emergency Medicine, Beth Israel Deaconess Medical Center and Harvard Medical School, Boston, MA USA; 4 Department of Health Policy and Management, Harvard T.H. Chan School of Public Health, Boston, MA USA; 5 Department of Medicine, Harvard Medical School, Boston, MA USA; 6 Division of General Internal Medicine, Department of Medicine, Brigham and Women’s Hospital, Boston, MA USA

**Keywords:** Machine learning, Health care economics

## Abstract

Recent health reforms have created incentives for cardiologists and accountable care organizations to participate in value-based care models for heart failure (HF). Accurate risk stratification of HF patients is critical to efficiently deploy interventions aimed at reducing preventable utilization. The goal of this paper was to compare deep learning approaches with traditional logistic regression (LR) to predict preventable utilization among HF patients. We conducted a prognostic study using data on 93,260 HF patients continuously enrolled for 2-years in a large U.S. commercial insurer to develop and validate prediction models for three outcomes of interest: preventable hospitalizations, preventable emergency department (ED) visits, and preventable costs. Patients were split into training, validation, and testing samples. Outcomes were modeled using traditional and enhanced LR and compared to gradient boosting model and deep learning models using sequential and non-sequential inputs. Evaluation metrics included precision (positive predictive value) at k, cost capture, and Area Under the Receiver operating characteristic (AUROC). Deep learning models consistently outperformed LR for all three outcomes with respect to the chosen evaluation metrics. Precision at 1% for preventable hospitalizations was 43% for deep learning compared to 30% for enhanced LR. Precision at 1% for preventable ED visits was 39% for deep learning compared to 33% for enhanced LR. For preventable cost, cost capture at 1% was 30% for sequential deep learning, compared to 18% for enhanced LR. The highest AUROCs for deep learning were 0.778, 0.681 and 0.727, respectively. These results offer a promising approach to identify patients for targeted interventions.

## Introduction

Annual U.S. costs for treating heart failure (HF) are estimated at nearly $30 billion; by 2030, about 3% of the population is expected to have HF, potentially increasing costs to $53 billion^1^. Considerable HF costs are potentially preventable through high-quality ambulatory care, particularly costs related to emergency department (ED) visits and hospitalizations for acute HF exacerbations^2,3^. Over the past decade, the increasing shift away from fee-for-service (FFS) to value-based models has led to a new financial environment where cardiologists and accountable care organizations (ACO) assume greater accountability for their patients' quality of care and preventable costs^4^.

Success in value-based models requires tools to facilitate identification of patients at high risk for preventable costs and utilization^2,4^. Such tools have traditionally employed statistical methods such as logistic or linear regression, which are limited as they tend to be unidimensional and focus on linking known predictors with outcomes. These models do not leverage the multifactorial effects of a large combinations of variables and do not account for dynamic change of variable over time^5^. By contrast, machine learning modeling is more suited to handle nonlinear risk prediction and complex interactions among predictors. These abilities have led to a growing interest in machine learning models in HF where combinations of clinical, demographic, and socioeconomic factors contribute to outcomes^5^. Deep learning, a subset of machine learning based on artificial neural networks, may be particularly useful in HF outcomes with demonstrated efficacy in areas where pattern recognition is necessary, as well as patient trajectory modeling, disease inference, and clinical decision support^6,7^.

Modeling of utilization in HF has traditionally focused on 30-day hospital readmissions, HF hospitalizations, high cost, and mortality^5,8–10^. A different approach is to focus solely on preventable outcomes. This approach may be more useful when the goal is to proactively identify patients with remediable utilization. To date, studies on preventable spending have assessed the trends, demographic disparities and geographic variation^2,3,11,12^. Yet, to our knowledge, only one study has attempted to predict preventable hospitalizations but did not focus specifically on HF and did not use machine learning^13^.

The goal of this study was to compare the performance of several deep learning approaches with traditional logistic regression in the prediction of preventable hospitalizations, ED visits, and costs using commercial claims data. We also describe the most prominent clinical factors that drive predictions.

## Methods

We used 12 years of U.S. commercial claims data to compare the predictive performance of traditional logistic regression modeling to deep learning models for the prediction of preventable hospitalizations, ED visits, and costs among HF patients. We used Python version 3.7 (Python Software Foundation) to perform analyses. Our study included completely de-identified data and therefore is not considered as a human subject research; a formal Institutional Review Board (IRB) review was not necessary. The study followed the Transparent Reporting of a Multivariable Prediction Model for Individual Prognosis or Diagnosis (TRIPOD) statement^14^.

### Data source

The study included 12 years of commercial claims data from a single large U.S. insurer between January 2006 and December 2017. The data encompassed all-payer insurance plans: individual, fully insured group, self-insured group, and Medicare Advantage plans. Diagnoses, procedures, and medications codes were grouped into a manageable number of clinically meaningful categories: ICD-10 (International Classification of Diseases 10th revision) diagnosis codes were grouped into CCS (Clinical Classifications Software) diagnosis categories^15^; CPT (Current Procedural Terminology) and ICD-10 procedure codes were grouped into CCS procedure categories^16,17^; National Drug Code (NDC) drug codes were grouped into RXIngredient and Anatomical Therapeutic Chemical (ATC) categories^18^. Annual costs were calculated as insurer payments for each patient in 2016 and 2017. To limit the impact of extreme outliers, we winsorized the top 1% to the 99th percentile.

### Study design and study population

**Figure 1:**
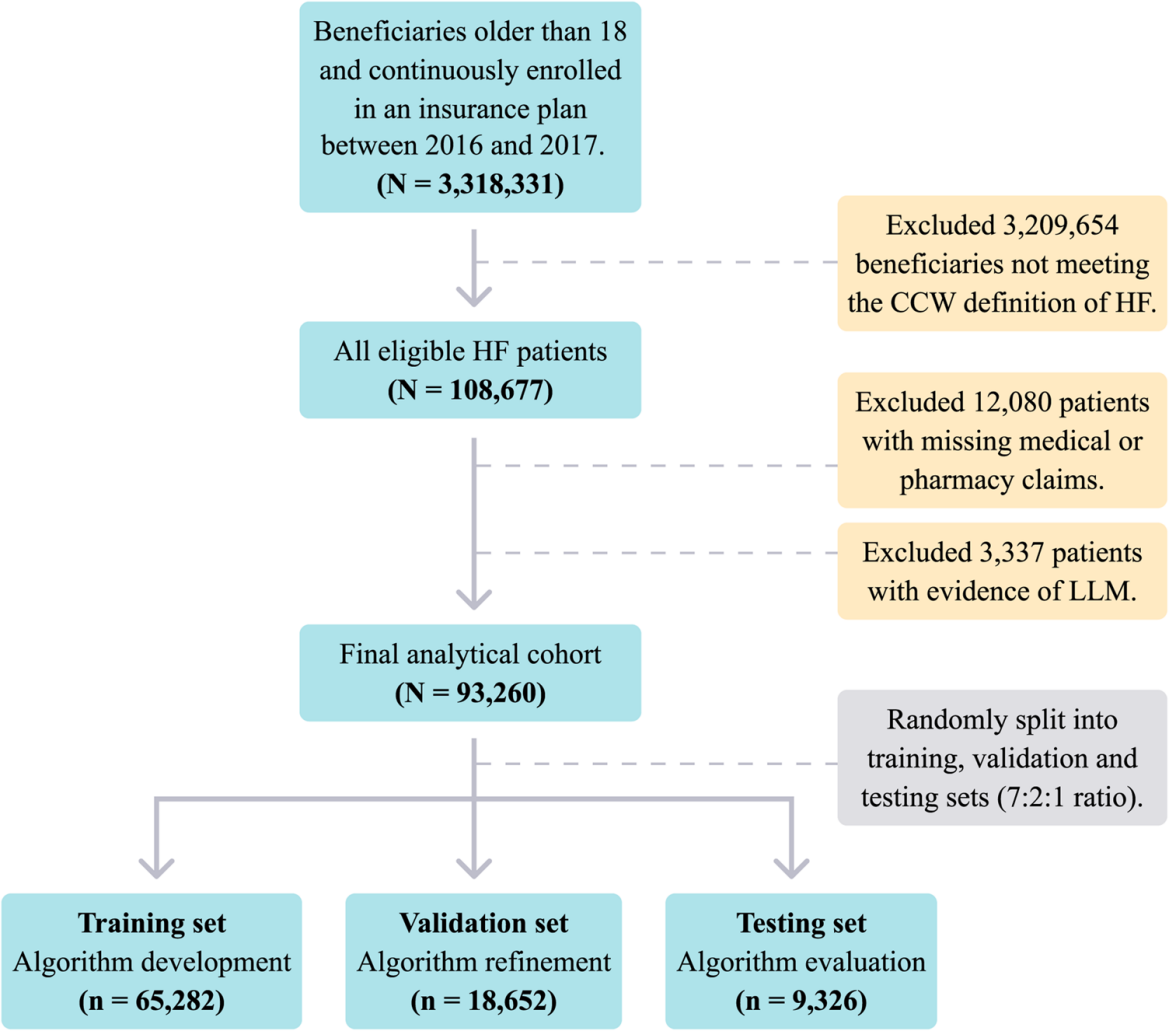
Patient Selection Process and the Creation of Training, Validation, and Testing Datasets. Patients were first defined as HF patients using the CMS' CCW algorithm. Next, patients with missing data or existing LLM were excluded. Finally, the cohort was randomly split using a 7:2:1 ratio into training, validation, and testing datasets. The figure was created using Adobe Illustrator Creative Cloud version 24.2.1^21^. CCW, Chronic Condition Warehouse; HF, Heart Failure; LLM, Life-Limiting Malignancies.

Data were divided into two time periods: an observation period (prior to 1/1/2017) and a prediction period (starting at 1/1/2017). We identified within observation period a cohort of HF patients aged 18 years or older using the CMS' Chronic Conditions Warehouse (CCW) algorithm^19^. We included HF patients continuously enrolled in an insurance plan between January 2016 to December 2017 with one or more medical and pharmacy claims (to ensure both were covered by their plan). In addition, we excluded patients with evidence of a life-limiting malignancy (LLM)^20^ within the observation window to avoid overestimating preventable costs. The process of patient selection is presented at Fig. 1.

### Outcomes

We focused on three binary outcomes. First, any preventable hospitalization within 6 months from 1/1/2017. Second, any preventable ED visit within 6 months from 1/1/2017. We chose 6 months as the prediction period because we reasoned that shorter-term utilization may be more clinically actionable. Third, any preventable costs, defined as costs of preventable hospitalizations and preventable ED visits within 1 year from 1/1/2017, which is a commonly used cost horizon.

To identify potentially preventable ED visits, we used a combination of two validated algorithms. The first was an updated version of an algorithm created by Billings et al.^22,23^ and validated by Ballard et al.^24^. It uses principal diagnosis codes to separate non-admitted ED visits into four categories: non-emergent; emergent but primary care treatable; emergent, ED care needed, but preventable; and emergent, ED care needed, and not preventable. We then used a second procedure-based algorithm, created by Moore et al.^25^, on the remaining unclassified visits to capture additional preventable visits where there was an absence of "ED-indicating procedures”.

To identify potentially preventable hospitalizations, we used the Agency for Healthcare Research and Quality (AHRQ) Prevention Quality Indicators (PQI)^26^. PQIs define potentially preventable hospitalizations, also known as ambulatory care sensitive conditions, as those related to conditions such as HF, diabetes, hypertension, and asthma, for which good outpatient care may prevent the need for hospitalization if provided in a timely manner. The algorithm has been validated and used in prior work^27^.

### Predictors

Patient features (i.e. predictors) were categorized as either knowledge-driven (i.e. traditional covariates) or data-driven (i.e. machine learning based covariates). Knowledge-driven features were constructed from patients' medical histories using a one-year observation window (i.e. January to December 2016). Data-driven features were constructed using either a 3- or 11-year observation window, as described below. A detailed description of the feature development process is presented in Online Table 1 and Online Fig. 1.

We manually compiled a set of 939 knowledge-driven features (based on domain experts' specifications) including demographics, episode counts and trends, hospital length of stay, readmission rates, costs, comorbidity indicators, major procedure indicators and chronic medications. Specific HF-related features included clinical subtypes, episodes, procedures, and HF medication adherence indicators.

Data-driven features are machine learning based representations of each patient's medical codes which serve as inputs for machine learning predictive models. We created these features using the Word2vec algorithm^28^, a natural language processing method which creates for each medical code in a patient's history a feature vector (i.e. an array of numbers representing each code). To form vectors representing each patient rather than a single code, patients' sets of vectors were summed and represented in two different approaches: single patient-level vectors (i.e. non-sequential vector inputs), containing the sum of a patient's 11-year medical history; temporal patient-level vectors (i.e. sequential vector inputs), containing 36 consecutive monthly vectors, each summing the medical codes for one month.

### Prediction models construction

We initially randomized our final cohort into training, validation and testing datasets using a 7:2:1 ratio (Fig. 1). Models were developed using the training set. The validation set was used to evaluate model fit on the training set while tuning model hyperparameters (i.e. model configuration parameters) and choosing the best performing models. Results are reported from the testing set.

We created five alternative prediction models for comparison (Table 1): two traditional LR models with a limited set of features, an enhanced LR model with a complete set of knowledge-driven features, and two machine learning models with different approaches. Traditional LR model 1 included the traditional features of age, gender and disease risk scores (CCS score^29^ and Chronic Condition Indicator (CCI) score^30^). In traditional model 2 we added cost features (inpatient, outpatient specialists, pharmacy, and primary care costs). These two models have been commonly used in risk scores in U.S. for diagnosis-based and pharmacy-based cost-prediction tools^31,32^. The enhanced LR model used the full set of 939 knowledge-driven features.

**Table 1:** Description of alternative traditional and enhanced risk prediction models.

Model	Model type	Feature description	Knowledge-driven feature count	Data-driven feature type
Traditional model 1	LR	Age, gender, disease risk scores	4	–
Traditional model 2	LR	Age, gender, disease risk scores, costs	14	–
Enhanced model	LR	Full knowledge- driven feature set	939	–
Non-sequential machine learning models	FNN, GBM	Full knowledge- driven feature set, data driven features	939	Single patient-level feature vectors
Sequential deep learning models	CNN, LSTM	Full knowledge- driven feature set, data driven features	939	Temporal patient-level feature vectors

CNN, Convolutional Neural Network, GBM, Gradient Boosting Model; LR, Logistic Regression; LSTM, Long Short-Term Memory.

Machine learning models were regarded as sequential or non-sequential, according to the models' input features. Machine learning models using non-sequential inputs (i.e. single patient-level vectors) included feedforward neural network (FNN) (Online Fig. 2) and gradient boosting model (GBM). Deep learning models using sequential inputs (i.e. temporal patient-level vectors) included Convolutional Neural Networks (CNN)^33^ and Long-Short Term Memory (LSTM)^34^ with an attention mechanism^35^ (Online Fig. 3). For each of the approaches, we chose the best performing models based upon the evaluation metrics. Additional information regarding the model development process is presented at Online Table 2.

### Evaluation methods and statistical analysis

Initially, we compared patient characteristics across the training, validation and testing samples using chi-square tests for categorical variables, Analysis of Variance (ANOVA) tests for continuous variables or corresponding nonparametric tests, as appropriate. Differences were considered statistically significant at *p* < 0.05. The 95% CIs were computed with 100 bootstrap replicates^36^.

Preventable hospitalizations and preventable ED visits were evaluated using the precision at k metric. Precision, which is also known as positive predictive value (PPV), is the proportion of patients predicted to have a preventable hospitalization or ED visits that actually have them.

Precision at k considers only the topmost patients (top k%) ranked by the model, and therefore presents the model's ranking accuracy at a specific threshold (we evaluated the thresholds between 1 and 10%). As an example, precision of 0.5 at k = 1% means 50% occurrence of an event among the patients ranked by the model at the top 1%. Preventable costs were evaluated using the cost capture metric^37^, defined as the ratio between the predicted preventable costs to actual preventable costs. This measure has been used to evaluate cost-prediction models in actuarial reports and health risk-assessment literature^32^. We reported the cost capture at k (i.e. the cost capture among the patients ranked by the model at the top k%) for the same thresholds. We also reported the Area Under the Receiver operating characteristic (AUROC) for all outcomes. Finally, we reported the features with the highest contribution to the model's performance. Because no standardized methods exist to identify individual important predictors from the deep learning models, we reported the top 15 important predictors from the GBM model.

## Results

### Patient characteristics

A total of 93,260 HF patients were identified and met inclusion criteria (Fig. 1), of which 65,282 were included in the training set, 18,652 in the validation set and 9326 in the testing set. Table 2 summarizes the baseline patient characteristics across cohorts. The overall cohort included 49.1% males, with an average age of 72 years. 67.8% of patients were enrolled in a Medicare Advantage program while the remainder in mostly fully insured and self-insured group plans. The average number of CCW comorbidities was 5.6 (the full CCW comorbidity list is presented at Online Table 3). The most prevalent comorbidities were hypertension (89.6%), hyperlipidemia (76.7%) and diabetes (42.4%). In the testing set, 9.1% of patients had either preventable hospitalizations or ED visits within the prediction period (1/1/2017–1/7/2017); 4.1% of patients had only preventable hospitalizations; 4.5% of patients had only preventable ED visits; 0.5% of patients had both preventable hospitalizations and ED visits. No significant differences were demonstrated between the training, validation, and testing sets.

**Table 2:** Baseline characteristics of the training, validation, and testing datasets.

	Training set (n = 65,282)	Validation set (n = 18,652)	Testing set (n = 9326)
Age*, mean (SD)	72 (11.9)	72.1 (11.9)	72.1 (11.8)
Male, %	48.9	48.9	50.1
Medicare Advantage beneficiaries^a^, %	67.7	67.9	67.9
US region*			
Northeast, %	15.1	15.5	15
Midwest, %	34.3	34.7	34.5
South, %	24.6	24	24.5
West, %	25.2	25	25.1
Comorbidities*			
Diabetes, No. (%)	36.6	37.5	36.3
Ischemic Heart Disease, %	48.8	49.3	49.4
Atrial fibrillation, No. (%)	23.7	23.9	23.9
COPD, No. (%)	17.3	17.5	17.3
Depression, No. (%)	11.5	11.3	11.2
Chronic kidney disease, No. (%)	32.4	32.8	32.6
Alzheimer’s disease, No. (%)	2.9	2.9	2.7
Hypertension, No. (%)	72.1	71.4	70.9
Hyperlipidemia, No. (%)	48.9	49.4	48.8
Number of CCW comorbidities*^,†^, mean (SD)	5.6 (2.8)	5.6 (2.8)	5.5 (2.9)
Chronic medications*^,‡^, mean (SD)	5.8 (3.5)	5.8 (3.5)	5.8 (3.5)
Annual total cost*, US$ median (IQR)	19,247 (10,925–38,274)	19,638 (11,171–39,212)	19,197 (11,032–37,706)
All hospitalizations per 1000 patients**	170.4	162.6	170.2
Preventable hospitalizations per 1000 patients**	52.2	51.7	51.8
All ED visits per 1000 patients**	178.4	180.5	179.3
Preventable ED visits per 1000 patients**	47.9	46.9	48.8
**Code counts per patient**^**§**^			
Diagnoses (CCS) per person, mean (SD)	210.6 (178)	208.8 (172.1)	209.3 (174.4)
Procedures (CCS) per person, mean (SD)	123.8 (116.5)	122.7 (112.7)	122.5 (111.9)
Medications (RxIngredient) per person, mean (SD)	270.1 (214.4)	272 (215)	269.8 (212.5)

*Results were calculated according to the observation period: 1/1/2016 – 12/31/2016.

^†^Among the CCW comorbidities evaluated at Online Table 3.

^‡^Defined as prescription drugs prescribed in the period of 7/1/2016–12/31/2016 as follows: at least one prescription of > 60 days, at least 2 prescriptions of 20 < days < 60. Oral, respiratory intravenous, intramuscular, and subcutaneous medications were included.

^§^ The number of codes is important when comparing deep learning algorithms. The number represents each patient's sum of codes throughout all of his history in the data.

** Calculated within the prediction period (1/1/2017–1/7/2017).

CCS, Clinical Classifications Software; CCW, Chronic Condition Warehouse (CCW); COPD, Chronic Obstructive Pulmonary Disease; ED, Emergency Department.

### Model predictive performance

Of the five candidate modeling approaches evaluated, the sequential deep learning models consistently provided the best predictive performance across all three outcomes and was closely followed by the non-sequential machine learning models (Table 3; Fig. 1). Highest AUROCs obtained for preventable hospitalizations, preventable ED visits and preventable costs were 0.778 (95% confidence interval [CI] 0.784–0.79), 0.681 (95% CI 0.68–0.685) and 0.727 (95% CI 0.725–0.728), respectively. These results represent an improvement in AUROC over traditional model 1 (age, gender, CCS and CCI scores) of 15.1, 11.4 and 13.9%, respectively.

**Table 3:** Discriminative performance comparison of alternative traditional and enhanced risk prediction models for the prediction of preventable hospitalizations, preventable emergency department visits, and preventable costs among heart failure patients.

	AUROC (95% CI)		
	Preventable hospitalizations	Preventable ED visits	Preventable costs
Traditional LR model 1	0.676 (0.674–0.677)	0.611 (0.608–0.614)	0.638 (0.637–0.640)
Traditional LR model 2	0.711 (0.709–0.713)	0.632 (0.630–0.635)	0.659 (0.657–0.661)
Enhanced LR model	0.747 (0.744–0.751)	0.652 (0.649–0.654)	0.718 (0.716–0.719)
Non-sequential machine learning models	0.770 (0.773–0.779)	0.665 (0.664–0.667)	0.726 (0.725–0.730)
Sequential deep learning models	**0.778** (0.784–0.790)	**0.681** (0.680–0.685)	**0.727** (0.725–0.728)

AUROC, Area Under the Receiver Operating Characteristic; CI, Confidence Interval; LR, Logistic Regression.

**Figure 2:**
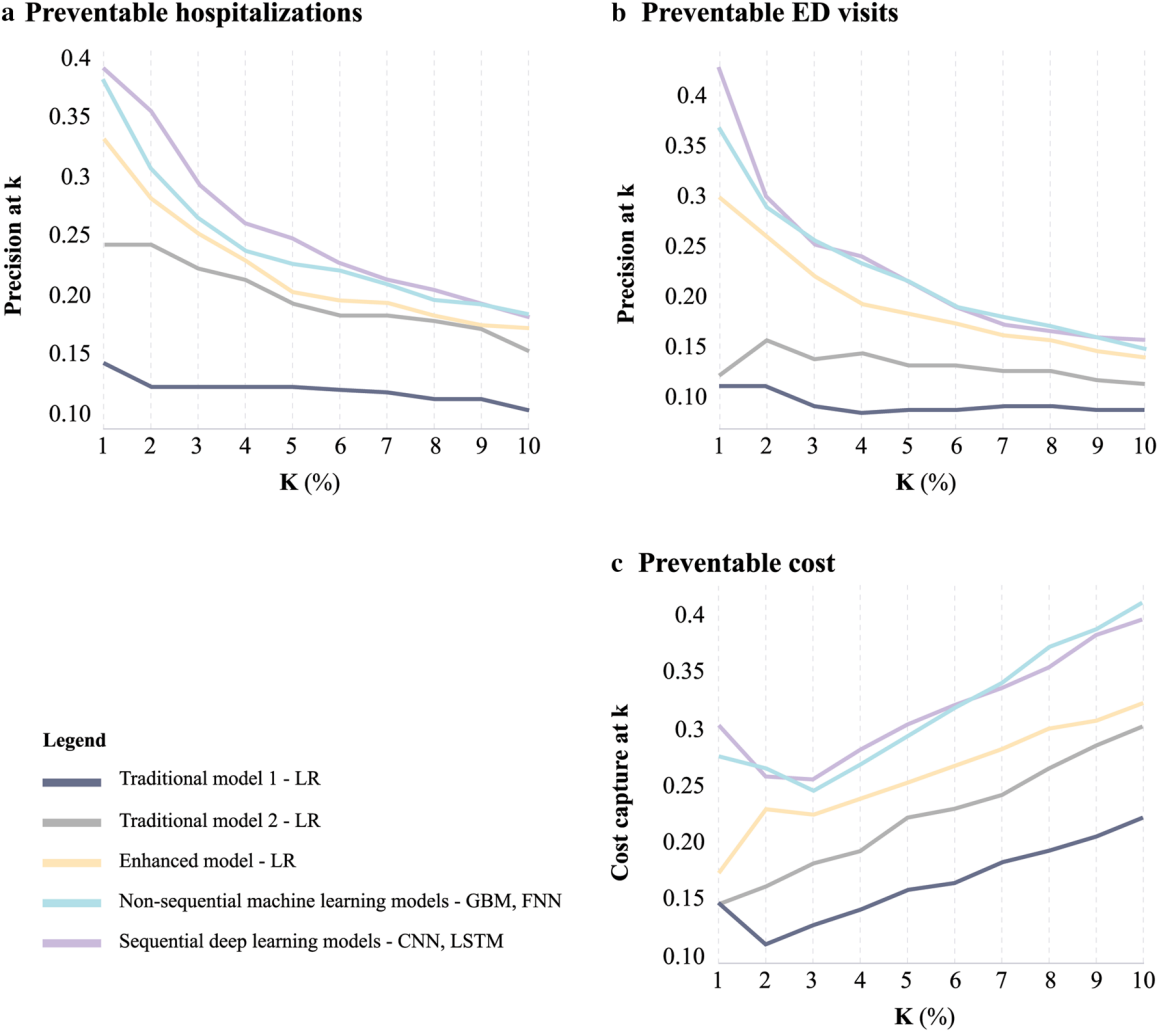
Predictive Models to Assess the Precision at k and Cost Capture at k for the Prediction of Preventable Hospitalizations, Preventable Emergency Department Visits, and Preventable Costs among Heart Failure Patients. (**a**, **b**) precision (PPV) at k for different thresholds (k). Precision at k considers only the topmost patients (top k%) ranked by the model, and therefore presents the accuracy of the model's ranking at a certain threshold. E.g. precision at 10% corresponds to PPV among the patients ranked by each model at the top decile. (**c**) cost capture at k for different thresholds (k), E.g. cost capture at 10 corresponds to the ratio between the predicted preventable costs to the actual preventable costs among the patients ranked by each model at the top decile. The figure was edited using Adobe Illustrator Creative Cloud version 24.2.1^21^. CNN, Convolutional Neural Network; FNN, Feedforward Neural Network; GBM, Gradient Boosting Model; LSTM, Long Short-Term Memory; LR, Logistic Regression; PPV, Positive Predictive Value.

Figure 2 depicts the superiority of deep learning models over LR when evaluating precision and cost capture for all thresholds between 1 and 10%. At k = 1% for example the sequential deep learning model demonstrated superiority over all other methods and reached a precision at 1% (PPV among patients ranked at the top 1%) of 39% and 43% for preventable hospitalizations and preventable ED visits, respectively (compared to 12% and 15% in traditional model 1); Cost capture at 1% was 30.1% for preventable costs (compared to 15% in traditional model 1). At k = 5%, the sequential deep learning model reached a precision at 1% of 26% and 21% for preventable hospitalizations and preventable ED visits, respectively (compared to 12% and 8.4% in traditional model 1); Cost capture at 5% was 30% for preventable costs (compared to 15.6% in traditional model 1).

### Feature contribution using GBM

**Figure 3:**
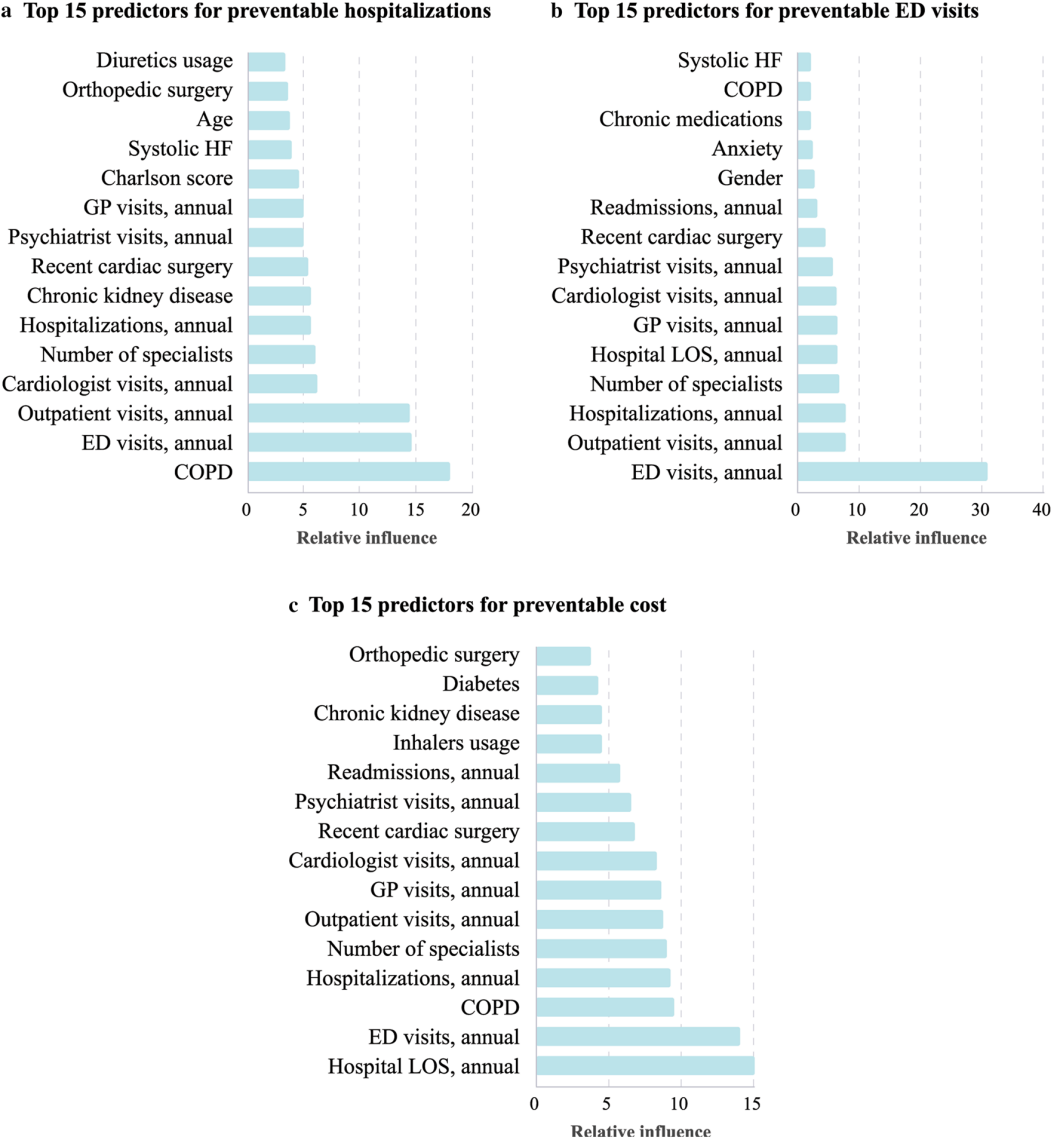
Most Influential Predictors Selected by the GBM for the Prediction of Preventable Hospitalizations, Preventable Emergency Department Visits, and Preventable Costs among Heart Failure Patients. Relative influence values range from 0 to 100 and indicate the relative contribution of a feature in predicting the outcome of interest. Relative influence from the GBM is plotted for the top 15 features of each of the three outcomes. The figure was edited using Adobe Illustrator Creative Cloud version 24.2.1^21^. LOS, Length of Stay; COPD, Chronic Obstructive Pulmonary Disease; ED, Emergency Department; GBM, Gradient Boosting Model; GP, General Practitioner; HF, Heart Failure.

We examined the 15 most influential features per outcome selected by GBM (Fig. 3). No feature vectors or costs were included in the analysis to focus solely on clinical features. Previous healthcare utilization features such as annual ED visits, outpatient visits, hospitalizations and LOS were selected across all outcomes with relative influence (RI) values ranging from 5.3 to 31.5. Chronic obstructive pulmonary disease (COPD) and chronic kidney disease were the most influential clinical comorbidities for preventable hospitalizations (RI of 17.7 and 5.3, respectively) and cost (RI of 9.1 and 4.2, respectively). For preventable ED visits, anxiety disorder was the most dominant comorbidity (RI = 2.4).

## Discussion

Novel payment models have increased the need for cardiologists to employ better prediction tools to identify preventable utilization for HF patients. We found that deep learning models outperformed traditional logistic regression models for HF outcomes with respect to discrimination, precision, and cost capture in identifying patients at highest risk for preventable utilization and cost. This suggests a promising role for deep learning in efficiently managing population health and HF costs.

Our main evaluation method for preventable hospitalizations and ED visits was precision at k. This method was adapted from the field of information retrieval^38^, where web search engines are a common use case. In web search, precision at k evaluates search results and corresponds to the proportion of relevant results among the top k percentile, as search engine users are mostly interested in the topmost retrieved results. Applying this same theory to HF patients, a cardiologist or an accountable care organization (ACO) may search for patients based on their risk of future preventable use or cost, with the goal of deploying limited resources, i.e. care management team, for interventions. Using this approach, assume in a population of 10,000 HF patients, resources exist for a care management program to be applied to 100 or 1% of a population. With LR results (Fig. 2), 12 in the top 100 would have preventable hospitalizations, while with sequential deep learning model would correctly predict 39 in 100. Cost capture at k evaluates the proportion of preventable costs captured by the model among the patients ranked by the model at the top k percentile. Similar to the previous example, assuming the top 1% of predicted patients received a care management program, this would potentially impact 15% of the preventable costs using LR, while with sequential deep learning model would impact 30.1%. Therefore, deep learning allows for better patient identification and potentially greater expected cost reductions with the same investment in resources.

Our study adds to a growing body of literature that compared machine learning methods to traditional models for HF (and other chronic conditions) outcomes. Desai et al.^5^ demonstrated superior predictive performance of GBM modeling over LR as well as random forest modeling in predicting HF hospitalizations. Using claims data only, they reported an AUROC of 0.745, which was lower than our study (0.778) for overall preventable hospitalizations. Other work by O’Donovan et al.^39^ that used claims data and more granular clinical data from EHRs similarly reported an AUROC of 0.8 on the prediction of unplanned HF admissions. Inclusion of detailed clinical data to our deep learning models may therefore This suggests that the deep learning models can be improved on if health systems and clinical leaders can augment their claims data on patients with detailed EHR data.

Another study by Min et al.^40^ compared several deep learning methods with LR for 30-day readmissions among COPD patients, finding no significant improvement in prediction with deep learning. This means that deep learning may be more suited to predict certain types of outcomes – perhaps with longer prediction periods rather than shorter ones; however, future study will be needed to determine which types of health outcomes are best predicted by deep learning.

Nevertheless, there are criticisms of deep learning, the main one being the lack of model interpretability. Much like search results, these models are often regarded as "black boxes", where only the input and output are clear^6^. To illustrate the main drivers for each outcome, we conducted GBM with solely clinical predictors as inputs (Fig. 3). We found that prior healthcare utilization was the dominant predictor for all three outcomes, along with several comorbidities such as COPD, chronic kidney disease (CKD), and anxiety disorder. Our results were largely similar to those of Desai et al. for HF hospitalizations using claims data. Yet, the addition of electronic health record (EHR) data by Desai also added important predictors such as laboratory and echocardiography results. This suggests that applying deep learning methods with more detailed EHR data, if available, may not only improve the predictive performance but also improve the clinical interpretability of the results^5^.

### Strengths and limitations

There are some key strengths of our study. First, we focused on a set of non-traditional preventable outcomes defined by validated algorithms. Previous studies dealing with preventable utilization and spending mainly evaluated the aspects of trends, demographic disparities and geographic variations^2,3,11,12^. Second, we suggest a novel approach for the definition of preventable ED visits (and consequently preventable costs) which takes into account unclassified cases by the Billings algorithm. We believe that this modified definition provides a more accurate estimation of preventable costs. Third, we used a large data sample which is crucial to properly train deep learning models.

Our study also has several limitations. First, working with de-identified claims data, our data did not include key information such as socioeconomic data (race, marital status, income level etc.), laboratory results and other EHR data which have shown to be strong predictors of HF readmissions and mortality^41^. This information was not available in the commercial data for analysis. Second, although our definition of preventable hospitalization has been well-validated previously, not all hospitalizations identified by the algorithm are necessarily preventable. There also may be other hospitalizations that may have been preventable that were not classified as so by the PQIs. Studies focusing on broadening and sharpening the current definitions of preventable visits may help researchers in honing predictions. Third, working on a single dataset, our results were internally validated with a random split of validation and testing datasets and did not include external validation. Further analyses should include external validation on a separate dataset. Fourth, while it is possible that the results may be marginally different depending on the ratio use for the training, validation, and testing tests, we chose the ratio of 7:2:1 based on prior literature using data science approaches^41^. Fifth, we focused our main analysis on precision at k from 1 to 10% given that this is a commonly used threshold in the literature to define high-need, high-cost populations, including in a recent report by the National Academy of Medicine^42^, and considered a clinically actionable range for a population health approaches to target individuals at highest risk. Although further analysis at higher precision cutoffs may yield different results, we think that is unlikely given the clear trends in Fig. 2 with lower performance at higher k percentiles. Sixth, we used a large amount of longitudinal data (11 years). Given more recent data are likely of greater importance because of the progressive nature of heart failure, and thus more likely follow a Markov process, it is possible similar results could have been obtained using less data, which may be more efficient to implement in practice. Future work should examine the relationship between data volume / timing and model performance . dataset. Fourth, while it is possible that the results may be marginally different depending on the ratio use for the training, validation, and testing tests, we chose the ratio of 7:2:1 based on prior literature using data science approaches^42^. Fifth, we focused our main analysis on precision at k from 1 to 10% given that this is a commonly used threshold in the literature to define high-need, high-cost populations, including in a recent report by the National Academy of Medicine^43^, and considered a clinically actionable range for a population health approaches to target individuals at highest risk. Although further analysis at higher precision cutoffs may yield different results, we think that is unlikely given the clear trends in Fig. 2 with lower performance at higher k percentiles. Sixth, we used a large amount of longitudinal data (11 years). Given more recent data are likely of greater importance because of the progressive nature of heart failure, and thus more likely follow a Markov process, it is possible similar results could have been obtained using less data, which may be more efficient to implement in practice. Future work should examine the relationship between data volume/timing and model performance.

## Conclusions

Our study suggests that deep learning methods have superior predictive performance over traditional statistical methods in identifying patients at high risk for preventable outcomes. Therefore, cardiologists and health system leaders should consider employing deep learning techniques to identify patients with potentially preventable acute care events and use these approaches to tailor targeted interventions to reduce unnecessary spending.

## Supplementary Information

Supplementary Figure 1.

Supplementary Figure 2.

Supplementary Figure 3.

Supplementary Information.

## Data Availability

The data that support the findings of this study originate from a large U.S. insurer. Restrictions apply to the availability of these data and they are therefore not publicly available. Due to restrictions, these data can be accessed only by request to the authors.

## Code availability

The code used in this study for the architecture of the machine learning models is available at https://github.com/DiagnosticRobotics/dr-chf-models.
