# Supplementary figures and images for: Comparison of deep learning with traditional models to predict preventable acute care use and spending among heart failure patients
Source: Sci Rep. 2021 Jan 13;11:1164. doi: 10.1038/s41598-020-80856-3 (PMC7806727; doi:10.1038/s41598-020-80856-3)

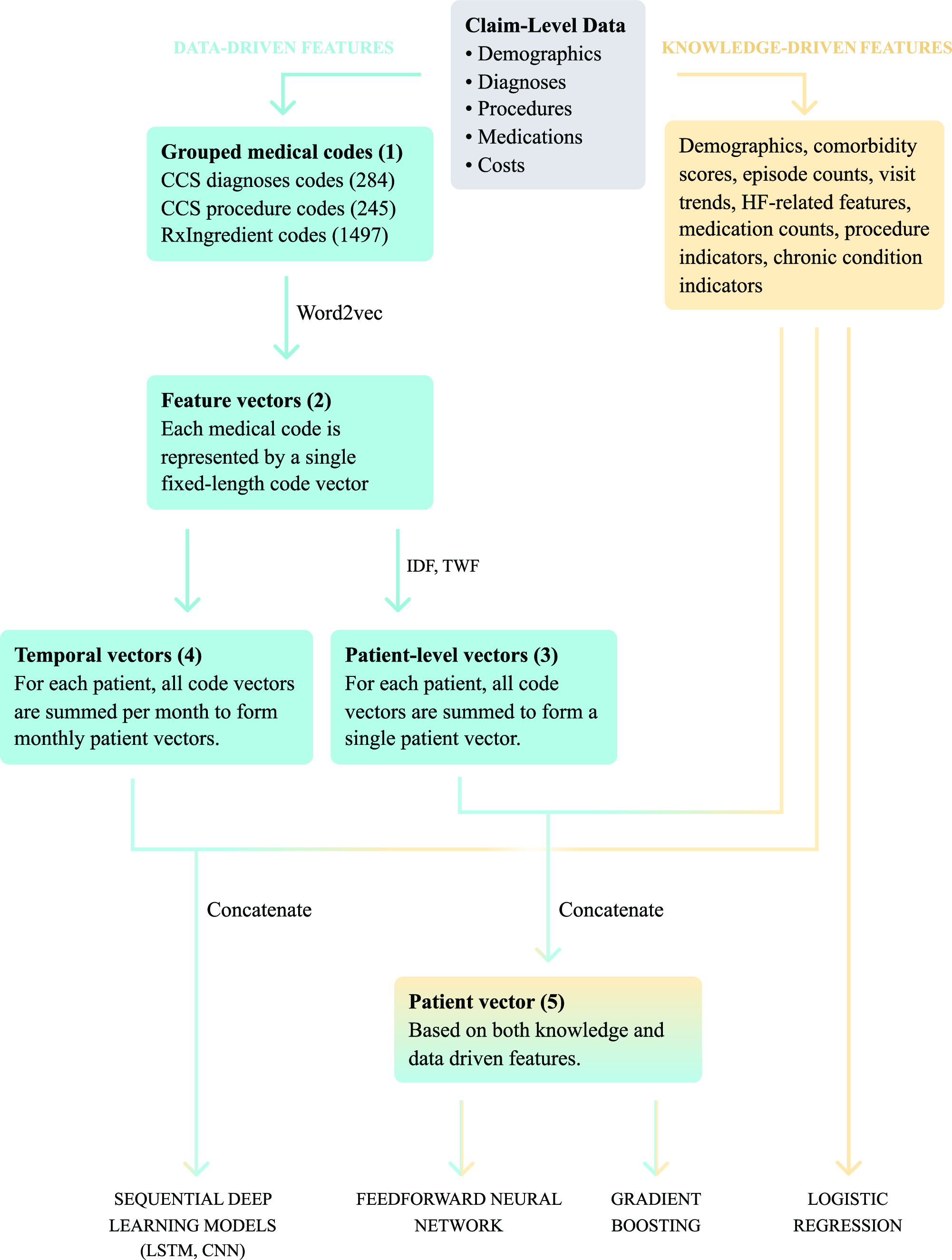

Supplement: Supplementary file 1 — Supplementary Figure 1. [file 41598_2020_80856_MOESM1_ESM.tif]

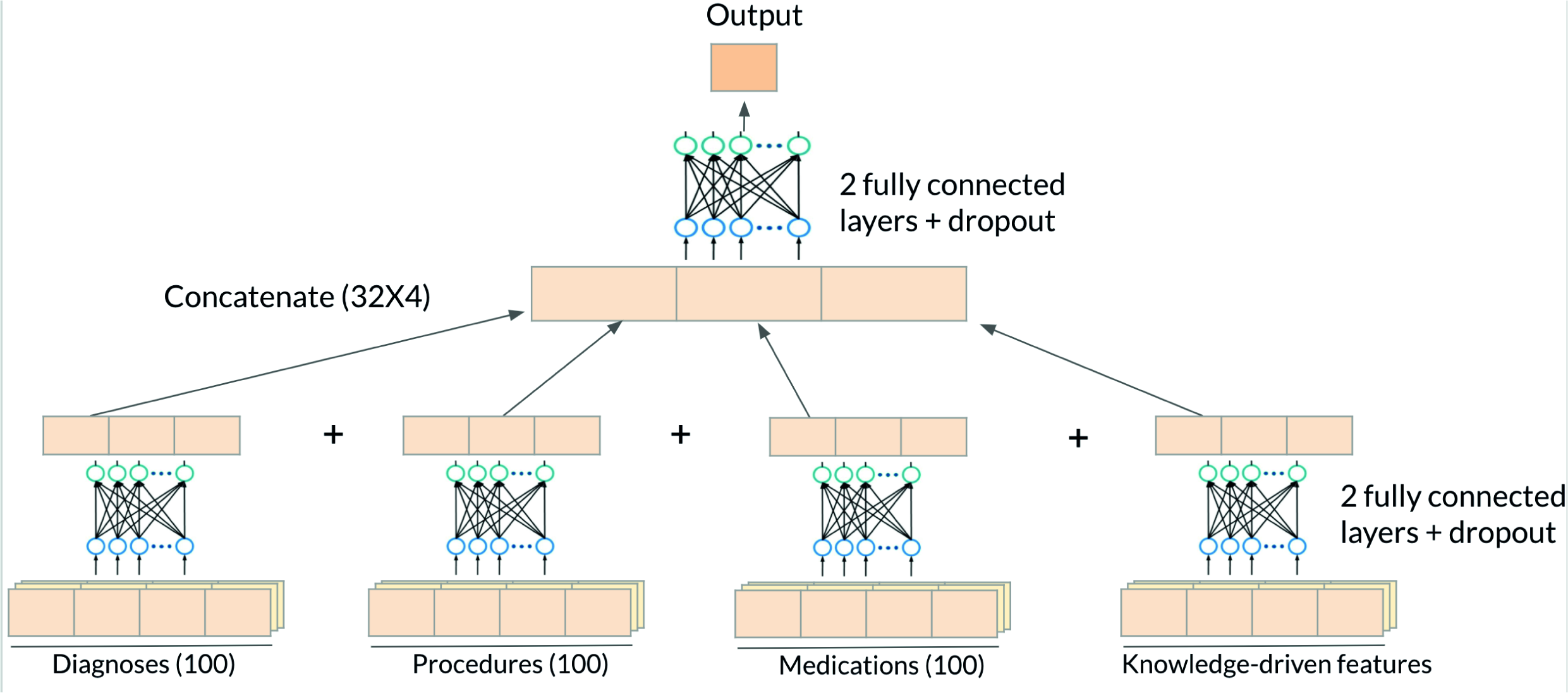

Supplement: Supplementary file 2 — Supplementary Figure 2. [file 41598_2020_80856_MOESM2_ESM.tif]

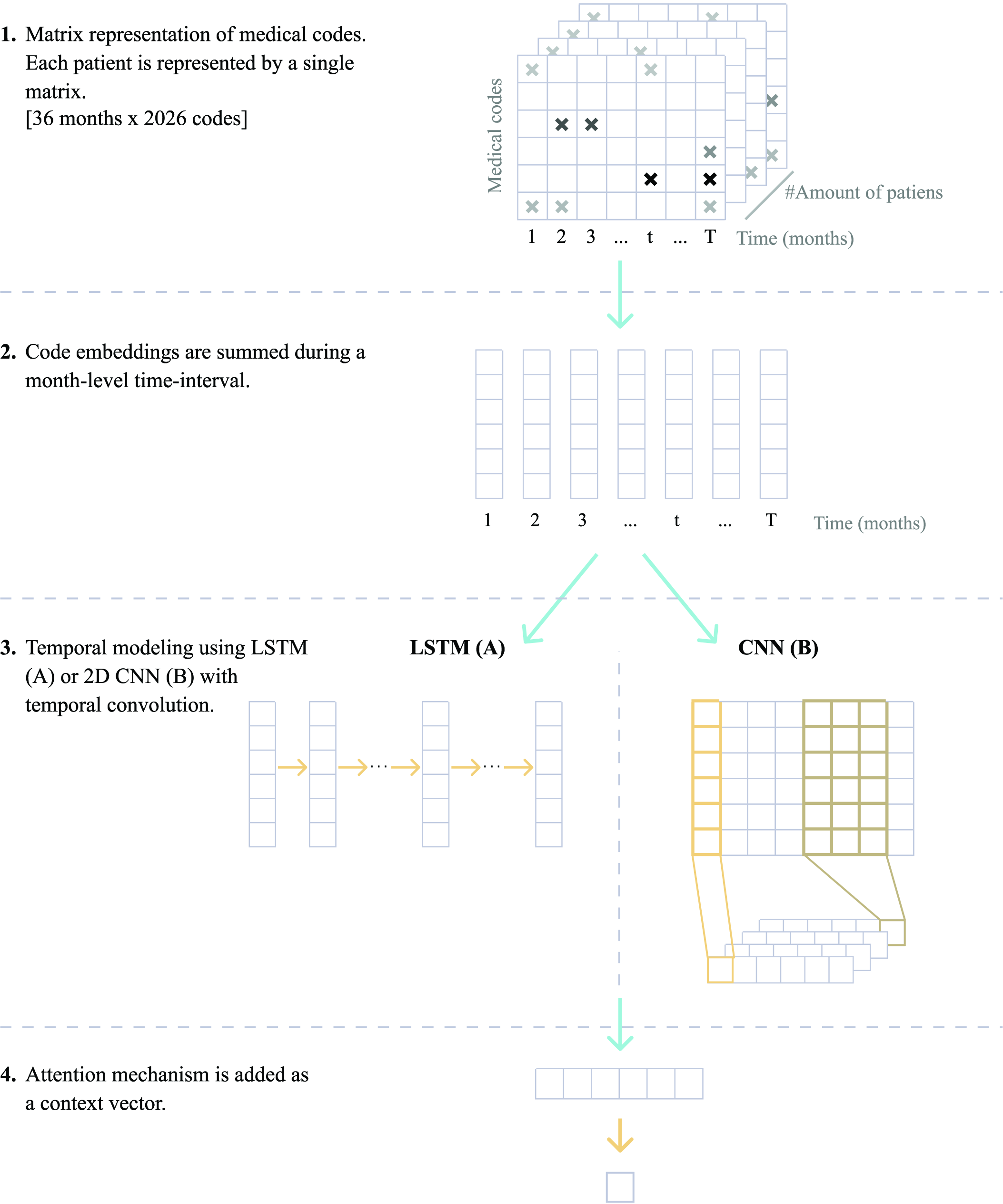

Supplement: Supplementary file 3 — Supplementary Figure 3. [file 41598_2020_80856_MOESM3_ESM.tif]
