# Supplementary material for: Comparison of deep learning with traditional models to predict preventable acute care use and spending among heart failure patients
Source: Sci Rep. 2021 Jan 13;11:1164. doi: 10.1038/s41598-020-80856-3 (PMC7806727; doi:10.1038/s41598-020-80856-3)
Supplement: Supplementary file 4 — Supplementary Information. [file 41598_2020_80856_MOESM4_ESM.docx]

**Comparison of Deep Learning with Traditional Models to Predict** **Preventable Acute Care Use and Spending among Heart Failure Patients**

Maor Lewis M.D.^a*^, Guy Elad M.Sc.^a^, Moran Beladev M.Sc.^a^, Gal Maor M.Sc.^a^, Kira Radinsky PhD^a^, Jesse M. Pines, M.D., M.B.A., M.S.C.E. ^a,b^, Nathan l. Shapiro, M.D. ^a,c,f^, Jose F. Figueroa, M.D., M.P.H. ^d,e,f*^

1. Diagnostic Robotics Inc.
2. US Acute Care Solutions, Canton, OH, United States
3. Department of Emergency Medicine, Beth Israel Deaconess Medical Center and Harvard Medical School, Boston, MA, United States
4. Harvard T.H. Chan School of Public Health, Department of Health Policy and Management, Boston, MA, United States
5. Harvard Medical School, Department of Medicine, Boston, MA, United States
6. Brigham and Women's Hospital, Department of Medicine, Division of General Internal Medicine, Boston, MA, United States

Supplementary Material

- **Online Table 1**. Definitions and Development of Knowledge-driven and Data-driven Features
- **Online Table 2**. Description of Modeling Approaches
- **Online Table 3**. Distribution of CCW Conditions among Heart Failure Patients in the Training, Validation, and Testing Datasets
- **Online Figure 1**. Developmental Process of Knowledge-driven and Data-driven Features

**Caption**: A set of 939 knowledge-driven features was compiled by domain experts. Data-driven features were constructed using sets of grouped diagnosis, procedure, and medication codes. A total of 284 CCS diagnosis codes, 245 CCS procedure codes and 1497 RxIngredient medication codes were used in the process (step 1). We used the Word2vec algorithm to create a code vector for each medical code. Next, code vectors were summed into patient-level vector representations in two different architectures (steps 3-4). First, all code vectors in a patient's 11-year history were summed to form a single patient-level vector (step 3). Second, all code vectors were summed per patient per month (until a history of 36 months) to form 36 monthly patient-level vectors as an input for the sequential deep learning models (step 4). During both processes, two types of weights (Inverse Document Frequency (IDF) and temporal weighting function (TWF)) were added per code. The figure was created using Adobe Illustrator Creative Cloud version 24.2.1.

Abbreviations: CCS, Clinical Classifications Software; CNN, Convolutional Neural Network; HF, Heart Failure; LSTM, Long Short-Term Memory.

- **Online Figure 2**. Feedforward Neural Network (FNN) Architecture

**Caption**: Inputs for the FNN model consisted of three feature sets (diagnoses, procedures, medications) as well as the knowledge-driven features. For each feature set, we created a separate multiple hidden layer of size [64,32] with a dropout rate of 0.5. Then, we created a concatenated layer of size (32X4=128), which was further used as an input for two other fully connected layers of size [32,16] (with a dropout rate of 0.5 as well). Lastly, we used the Softmax activation function to map the non-normalized results of the output layer into a probability distribution. The figure was created using Adobe Illustrator Creative Cloud version 24.2.1.

- **Online Figure 3**. Sequential Deep Learning Models Development

**Caption**: Each patient was represented as a matrix of medical codes on a monthly based interval during each patient's 36 months history (step 1(. Next, all code vectors within each month were summed to form a single vector (step 2). Then, LSTM and CNN sequential architectures were applied. CNN model (step 3A) consisted of 2D convoluted layers, kernel size of 3 and 6 months, 100 filters and max pooling for each layer. LSTM model (step 3B) consisted of two LSTM layers of size [64,32]. The LSTM model's output together with the knowledge-driven features were inserted into two fully connected layers. Attention mechanism (step 4) was added on top of the CNN and LSTM models to highlight each patient's most important parts of his medical history. The figure was created using Adobe Illustrator Creative Cloud version 24.2.1.

Abbreviations: CNN, Convolutional Neural Network; LSTM, Long Short-Term Memory.

| **Online Table 1**. Definitions and Development of Knowledge-driven and Data-driven Features | |
| --- | --- |
| Knowledge-driven features | We manually compiled a set of 939 knowledge-driven features including demographics (age, gender, US region), episode counts and trends (ED visits, hospitalizations, outpatient specialist visits, general practitioner visits), hospital length of stay and readmission counts, costs (ED, outpatient specialists, hospitalizations, pharmacy), chronic conditions indicators defined by the CCW algorithms (19), comorbidities scores: Charlson score (41), CCS score (28), CCI score (29), major procedure indicators, chronic medications counter. HF-related features included HF subtype indicators (diastolic, systolic, hypertensive), HF-related episodes counts and trends (ED visits, hospitalizations, cardiologist visits, cardiac echography), cardiac echography counts, implantable cardioverter-defibrillator or cardiac resynchronization therapy (CRT) indicator, non-adherence indicator (42) for HF medications (ACE-inhibitors\ARBs, Beta Blockers, digoxin, loop diuretics, nitrate, hydralazine). |
| Data-driven features | Data-driven features are representations of each patient's set of medical codes throughout their history and serve as an input for the machine learning predictive models. The process is presented in Online Figure 1. Features were constructed using sets of grouped diagnosis, procedure, and medication codes. A total of 284 CCS diagnosis codes, 245 CCS procedure codes and 1497 RxIngredient medication codes were used in the process (step 1 in Online Figure 1). Initially, inspired by the well-known Word2vec algorithm (27) (a natural language processing methods which assigns for each word in a sentence a vector representation), we created code vectors for each medical code, using continuous bag of words (CBOW) with negative sampling (step 2 in Online Figure 1). The idea was to treat a patient's set of medical codes as if it was a sentence consisting of words. Next, code vectors were summed into patient-level vector representations in two different architectures (steps 3-4 in Online Figure 1). First, all code vectors in a patient's 11-year history were summed to form a single patient-level vector (step 3 in Online Figure 1). Second, all code vectors were summed per patient per month (until a history of 36 months) to form 36 monthly patient-level vectors as an input for the sequential deep learning models (step 4 in Online Figure 1). During both processes, two types of weights were added per code. The first was Inverse Document Frequency (IDF), which grants higher impact to less frequent codes than frequent ones and thus reduces the impact of frequently used administrative codes for example. The second was a temporal weighting function (TWF), which takes into consideration the time interval between the code's date and the prediction date. In that way recent codes have more impact than the previous ones. |

| **Online Table 2**. Description of Modeling Approaches | |
| --- | --- |
| Model | Description |
| Logistic Regression (LR) | Logistic regression is a commonly used model in applied health services research. It can be used to explain the relationship between one dependent binary variable and one or more independent variables. We applied two regularization techniques: Ridge Regression, which penalizes the sum of squared coefficients (L2 penalty), and Least Absolute Shrinkage and Selection Operator (LASSO) Regression, which penalizes the sum of absolute values of the coefficients (L1 penalty). |
| Gradient Boosting Model (GBM) | GBM is a tree-based ensemble learning method in which several weak classifiers are sequentially constructed and combined while correcting prediction errors made by the previous ones to form a strong learner. We used the XGBoost implementation, and tuned the maximum depth of a tree, minimum split loss, subsample ratio of the training instances per tree, subsample ratio of features per tree, learning rate, and the number of trees. |
| Feedforward Neural Network (FNN) | FNN is a traditional deep learning method characterized by a unidirectional decision flow advancing from the input to the output in successive layers. We computed patient vector representations separately for diagnoses, procedures, and medications, thus creating three different feature sets (The final patient representation is presented in equation1). The FNN model's input consisted of these three feature sets as well as the knowledge-driven features, as presented in Online Figure 2. For each feature set, we created a separate multiple hidden layer of size [64,32] with a dropout rate of 0.5. Then, we created a concatenated layer of size (32X4=128), which was further used as an input for two other fully connected layers of size [32,16] (with a dropout rate of 0.5 as well). Lastly, we used the Softmax activation function to map the non-normalized results of the output layer into a probability distribution.  Equation 1: Patient Representation  $\tilde{f}_{i}= \frac{\sum_{j\in C_{i}} w_{j}\cdot V_{j}}{\sum_{j\in C_{i}} w_{j}},$  $w_{j}={IDF}_{j}\cdot\log\left( 1+\frac{1}{\Delta t_{j}} \right)$  $\tilde{f}_{i}$ is the feature representation of patient $i$, which is a weighted average of all the codes associated with the patient;$C_{i}$represents the medical codes of patient $i;$ $W_{j}$is the weight of code $j;$ $V_{j}$is the vector representation of code $j$ learned by Word2vec; ${IDF}_{j}$ is the inverse document frequency of code $j$ and $\Delta t_{j}$ is the delta time between code $j$ and prediction date (set as 1/1/2017). |
| Convolutional Neural Networks (CNN), Long-Short Term Memory (LSTM) | We constructed a set of sequential deep learning models, including CNN and LSTM. Each patient was represented as a matrix of medical codes on a monthly based interval during each patient's 36 months history (step 1 in Online Figure 3). Next, all code vectors within each month were summed in the same manner presented earlier under equation 1. Thus, each time interval was represented by a single vector (step 2 in Online Figure 3). Then, LSTM and CNN sequential architectures were applied:  CNN (step 3A in Online Figure 3)- consisting of 2D convoluted layers, kernel size of 3 and 6 months, 100 filters and max pooling for each layer.  LSTM (step 3B in Online Figure 3)– consisting of two LSTM layers of size [64,32]. The LSTM model's output together with the knowledge-driven features, were inserted into two fully connected layers.  Attention mechanism (step 4 in Online Figure 3) was added on top of the CNN and LSTM models to highlight each patient's most important sections of his medical history.  * For each of the models, we used sample weighting to handle unbalanced data. |

| **Online Table 3**. Distribution of CCW Conditions among Heart Failure Patients in the Training, Validation, and Testing Datasets | | | |
| --- | --- | --- | --- |
| CCW condition | Training set  (n= 65,282) | Validation set  (n= 18,652) | Testing set  (n= 9,326) |
| Diabetes, %  Ischemic Heart Disease, %  Acute Myocardial Infarction, %  Atrial Fibrillation, %  Asthma, %  Chronic Obstructive Pulmonary Disease, %  Stroke / Transient Ischemic Attack, %  Chronic Kidney Disease, %  Depression, %  Glaucoma, %  Hip / Pelvic Fracture, %  Hyperlipidemia, %  Hypertension, %  Osteoporosis, %  Rheumatoid Arthritis / Osteoarthritis, %  Cataract, %  Acquired Hypothyroidism, %  Alzheirmer’s Disease, %  Alzheirmer’s Disease, Related Disorders or Senile Dementia, %  Anemia, %  Benign Prostatic Hyperplasia, %  Cancer, Colorectal, %  Cancer, Endometrial, %  Cancer, Breast, %  Cancer, Lung, %  Cancer, Prostate, % | 36.6  48.8  2.9  23.7  5.6  17.3  5.6  32.4  11.5  10.5  1.3  48.9  72.1  3.6  31  14.2  13.5  2.9  8.4  21  6.5  0.9  0.1  2.6  0.05  2.3 | 37.5  49.3  3.1  23.9  5.9  17.5  5.7  32.8  11.3  10.5  1.3  49.4  71.4  3.5  31.6  14.7  13.4  2.9  8.4  20.5  6.6  0.9  0.2  2.4  0.06  2.2 | 36.4  49.4  3.2  23.9  5.7  17.3  5.9  31.5  11.2  10.3  1.1  48.8  70.9  3.6  31.1  15.1  12.7  2.6  7.7  20.2  6.7  0.8  0.1  2.7  0.06  2.3 |
| Abbreviations: CCW, Chronic Condition Warehouse (CCW). | | | |
